# Data-Driven-Based Approach to Identifying Differentially Methylated Regions Using Modified 1D Ising Model

**DOI:** 10.1155/2018/1070645

**Published:** 2018-11-18

**Authors:** Yuanyuan Zhang, Shudong Wang, Xinzeng Wang

**Affiliations:** ^1^School of Information and Control Engineering, Qingdao University of Technology, Qingdao, Shandong, China; ^2^College of Computer and Communication Engineering, China University of Petroleum (East China), Qingdao, Shandong, China; ^3^College of Mathematics and Systems Science, Shandong University of Science and Technology, Qingdao, Shandong, China

## Abstract

**Background:**

DNA methylation is essential for regulating gene expression, and the changes of DNA methylation status are commonly discovered in disease. Therefore, identification of differentially methylation patterns, especially differentially methylated regions (DMRs), in two different groups is important for understanding the mechanism of complex diseases. Few tools exist for DMR identification through considering features of methylation data, but there is no comprehensive integration of the characteristics of DNA methylation data in current methods.

**Results:**

Accounting for the characteristics of methylation data, such as the correlation characteristics of neighboring CpG sites and the high heterogeneity of DNA methylation data, we propose a data-driven approach for DMR identification through evaluating the energy of single site using modified 1D Ising model. Applied to both simulated and publicly available datasets, our approach is compared with other popular methods in terms of performance. Simulated results show that our method is more sensitive than competing methods. Applied to the real data, our method can identify more common DMRs than DMRcate, ProbeLasso, and Wang's methods with a high overlapping ratio. Also, the necessity of integrating the heterogeneity and correlation characteristics in identifying DMR is shown through comparing results with only considering mean or variance signals and without considering relationship of neighboring CpG sites, respectively. Through analyzing the number of DMRs identified in real data located in different genomic regions, we find that about 90% DMRs are located in CGI which always regulates the expression of genes. It may help us understand the functional effect of DNA methylation on disease.

## 1. Introduction

DNA methylation is an important epigenetic modification which plays an essential role in gene expression [1, 2] and cancers [3–5]. Aberrant methylation status, such as hypermethylation in promoter, often leads to gene silencing. It is an important mechanism of antioncogene inactivation [6]. Global hypomethylation always leads to the emergence of cancers through affecting the stability of chromatin [7]. There are pieces of evidence showing that abnormal methylation patterns are related to many cancers and other diseases [8–13]. Also, some genomic regions have been found instable in methylation, which increases methylation variability in cancer and then causes cancer heterogeneity [14–16]. Therefore, identification of aberrant methylation patterns is important to understand the pathogenesis of diseases.

With the development of high-throughput technologies, there are two main technologies to quantify genome-wide DNA methylation, bisulfite microarray, and sequencing which provide great opportunities for revealing the epigenetic mechanisms of diseases. Array technologies, Illumina Infinium HumanMethylation 27 K and 450 K, are often used to study complex diseases owing to their low cost and high-resolution ratio popularly. There are two designs of data form, *β*-values and* M-*values, used in identifying aberrant methylated patterns. *β*-value measures the proportion of methylated intensities out of total intensities, and* M-*value is calculated as the log2 ratio of the intensities of methylated probe versus unmethylated probe. The relationship between *β*-value and* M*-value is shown as the following equation:

(1) $$\begin{array}{c} M=log_{2}\left(\;\frac{\beta }{1-\beta }\;\right). \end{array}$$

It is shown that* M*-value may have more statistically valid for differential analysis of methylation [17].

Nowadays, various approaches have been proposed on the basis of microarray data to extract DNA methylation patterns, including differentially methylated loci [18–26] and differentially methylated region (DMR) [27–36]. Existing DMR detecting methods always consider some data characteristics to develop different assumptions. Bump hunter [27], DMRcate [28], and ProbeLasso [29] were developed through hypothesizing that the mean difference in methylation level of different groups is a primary cause in DMR identification; therefore, DMRs are identified through considering difference of mean signal between normal and cancer samples. Considering the heterogeneity of cancer samples [37], Wang et al. [30] developed an approach based on integrating mean and variance signal to identify DMRs. It is noteworthy that DMR methods integrating more information, such as mean and variance signals, always have better performance than those integrating less information, such as considering only mean signal [30]. Therefore, considering that additional characteristic, highly correlated neighboring CpG sites [38] in methylation data are rarely integrated in existing DMR methods; a data-driven method is developed based on integrating more information of data.

Motivated by Ising model which describes matter phase transition considering the strong interaction among neighbor molecular, we consider DNA methylation in genome by 1D (one-dimension) Ising model which can integrate the effect of neighbor sites. For each site, the status depends on its differential significance (p-value) and that of their neighbors with correlation characteristic. Generally, if the status of the site is significant, the more the accordance of the neighbor sites with the site, the lower the energy. If the status of the site is nonsignificant and those of its neighbors are significant, there are strong correlation between the site and its neighbors; we think that the site may also have low energy by integrating all information. The reason is that methylation level of a site is affected by multiple factors expect for disease; the information of neighbor sites can amend the bias of the site caused by other confounders. DMRs are identified as regions with low energy.

## 2. Material and Methods

**Figure 1:**
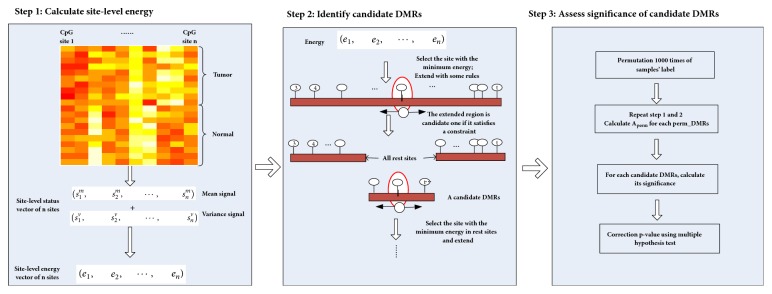
Flowchart of the proposed approach. Step 1, single site-level energy is calculated based on modified 1D Ising model. Step 2, candidate DMRs are identified using a greedy algorithm. Step 3, for each candidate DMR, the significance is assessed through permuting the sample labels.

We develop a data-driven approach to detecting DMRs (see Figure 1) which considers the data characteristics, correlation of neighboring CpG sites, and the high heterogeneity.

**Step 1 (calculate site-level energy).**

Motivated by the principle of 1D Ising model, we define the site-level energy as follows:

(2) eif=−∑j≠iJij·sif+sjf,sif=−logpvalueif,pvalueif≤0.050,pvalueif≥0.05,

where *e*_*i*_^*f*^ represents the energy of site *i* in feature *f*; *J*_*ij*_ represents the correlation of sites *i* and *j* in normal samples; and *s*_*i*_^*f*^ describes the signal value of *i* which represents the difference between tumor and normal samples in feature *f*. *pvalue*_*i*_^*f*^ in function (2) is used to describe whether the site *i* is significantly different between two groups under the feature *f*. Here, if p-value is less than 0.05, we believe that this site can provide energy to distinguish the two types of samples under this feature. Otherwise, no energy is provided. The smaller the p-value is, the more the energy it provides. Therefore, we use the negative log of *pvalue*_*i*_^*f*^ for energy when p-value is less than 0.05; otherwise, zero is used.

*pvalue*_*i*_^*f*^ is calculated using two paired* t*-tests and one-sided* Pitman-Morgan* test to describe the mean and variance signals, respectively, which are often used in other works [30]. Considering high heterogeneity of methylation in cancers, we integrate the mean and variance signal to define site-level energy by parameter *λ*; that is, *f* ∈ {*mean*, *variance*}. For mean and variance signals, the statuses are denoted by *s*_*i*_^*mean*^ and *s*_*i*_^*var*^, respectively. Therefore, the site-level energy is denoted as follows:

(3) $$\begin{array}{c} e_{i}=\lambda \cdot e_{i}^{mean}+\left(\;1-\lambda \;\right)\cdot e_{i}^{var}, \end{array}$$

where

(4) eimean=−∑j≠iJij·simean+sjmeaneivar=−∑j≠iJij·sivar+sjvarλ=eimeaneimean+eivar.

*λ* represents the weight of mean signal to total signals. For each site, the lower the energy is, the more possible the difference site between case and control is.

**Step 2 (identify candidate DMRs).**

To identify candidate DMRs, we define the total energy *E* of region *R*_*k*_ as follows:

(5) $$\begin{array}{c} E_{R_{k}}=\underset{i\in R_{k}}{\sum }e_{i}. \end{array}$$

We use a greedy algorithm with the following steps to identify candidate DMRs. Considering the question of what conditions of a site are required to add candidate regions, we use a permuted method. First, we permute the sample labels *n* times and calculate the permuted energy of each site using (3). Second, permuted energy of all sites is sequenced in ascending order and the value at 5% is selected as the threshold *τ*. Therefore, the greedy algorithm is executed in the following steps: (1) Select the CpG site with the lowest energy as a seed site if its energy is smaller than *τ*, which is considered as the starting regions *R*_*k*_. (2) Select the neighbor site of the current region with the lowest energy and add the site to *R*_*k*_ if energy of the site is smaller than *τ*. (3) Continue with the neighbors of *R*_*k*_ and keep adding the site to *R*_*k*_ until energy of the neighbor sites of *R*_*k*_ is greater than or equal to *τ*. (4) Choose another site with a seed one in the remaining sites except for *R*_*k*_ and repeat the above steps which can obtain another candidate region until the energy of any site of the rest is greater than *τ*.

**Step 3 (assess significance of candidate DMRs).**

To assess significance of candidate DMRs, we need to calculate p-value for each candidate region. We make the null hypothesis that a candidate region is not a DMR. If p-value is less than a significance level, the null hypothesis is rejected. The identification of DMR is equivalent to determining whether a candidate region is associated with the sample label. Therefore, p-value of a candidate region is calculated through permuting sample label. We complete the process of Step 2 for each permutation. For the *t*-th permutation, we can obtain *n*_*t*_ permuted DMRs. The energy of permuted DMR *R*_*perm*_*i*__ is denoted by *E*_*perm*_*t*_,*i*_, *i* = 1,…, *n*_*t*_. For each candidate DMR *R*_*k*_, the significance is measured by the p-value which is calculated as

(6) p=∑t=11000∑i=1ntIEpermt,i/sizeRpermi<ERk/sizeRk∑t=11000nt,

where *I*(*x*) is an indicator function and *size*(*R*) represents the numbers of CpG sites in region *R*. To consider the multiple testing, we use a function p.adjust in R and compare the results of different parameters; the results obtained with Bonferroni were the most conservative. Therefore, the p-values are multiplied by the number of comparisons using Bonferroni correction. The candidate DMR is considered significant if the p-value corrected by Bonferroni is smaller than 0.05.

## 3. Results and Discussion

In calculating the energy of each site, we hypothesized that a locus was associated with only two sites adjacent to its left and right for simplifying the correlation characteristic. To show the performance of the proposed method, we compare the method with bump hunting [27], DMRcate [28], ProbeLasso [29], and Wang's method [30] in simulation data. Applying to the real data, we compare the DMRs identified by our method and Wang's method [30] based on *β*-value and* M*-value, respectively. Also, based on* M*-value, we compare our method with DMRcate [28] and ProbeLasso [29]. Finally, the necessity of integrating characteristics of methylation data is analyzed.

### 3.1. Simulation Study

We generate simulation data referencing Wang's method [30] which consider the real characteristics of methylation data based on* M*-value. For case-control design, like Wang's method, we consider methylation measure *X* following a conditional scaled normal distribution:

(7) X ∣ Y=1,Z=z~z·Nμ,ΔTΣΔ,X ∣ Y=0,Z=z~z·N0,Σ,

where *z* ~ *Beta*(1, 1), *Y* = 1 and *Y* = 0 represent tumor and matched-control samples, respectively; the vector *μ* = (*μ*_1_, *μ*_2_,…,*μ*_*h*_)^*T*^ and Δ = (*v*_1_, *v*_2_,…,*v*_*h*_)^*T*^ represent mean and variance signals, respectively; an element Σ_*ij*_ in matrix Σ_*h*×*h*_ is *σ* × *ρ*^|*i* − *j*|^ which describes the correlation characteristic of neighboring sites *i* and *j*; and *h* is the number of consecutive sites in a defined cluster. In each simulation, we generate 100 tumor and control samples with 10000 methylation sites. The genomic positions of the 10000 sites are simulated by that of the first 10000 chromosome 1 on 450k array [30]. The clusters are obtained based on the genomic position of sites using R package “bump hunter”.

To show the performance of different methods, we select ten clusters randomly and set them as real DMRs as follows: for tumor samples, the first three are simulated mean signal only through setting *μ*_*i*_ in vector *μ* which follows the uniform distribution *unif*(*μ*_*a*_, *μ*_*b*_); the next three are simulated variance signal only through setting *v*_*i*_ = *α* + *ε* for tumor samples, where *α* is a basic value and *ε* is a random value following *unif*(0, 0.5); the last four are both simulated mean and variance signals through adding mean and variance signal in tumor samples by *μ*_*i*_ and *v*_*i*_. The sensitivity (SE) and specificity (SP) are defined according to the confusion matrix shown in Table 1:

(8) SE=dc+d,SP=aa+b.

We compare the performance of our method with bump hunting [27] and Wang's method [30] in simulated data based on different values of different parameters (see Table 2). To improve the significance, we implement 10 times for each set of parameters and calculate the mean values of specificity and sensitivity. It is shown that our method has higher sensitivity than other methods (see Table 3).

Furthermore, to avoid the deviation of the numbers of identified DMRs, the numbers of true positive (TP) and false positive (FP) DMRs are compared through calculating the overlap between identified DMRs and embedded true DMRs (see Table 3).

In this study, an identified DMR is known as a true positive one if the intersection of the DMR and some true DMR contains more than *L* CpG sites. *L* is defined by multiplying the length of the true DMR by *θ*. The greater the *θ* is, the higher the degree of overlap between the true positive DMR and the real area is. It is also shown that our method has higher matching degree with true DMRs when either *θ* = 0.2 or *θ* = 0.5. More simulations studies, changing simulation parameters, are described in supplementary material (see Tables S1-S3 for comprehensive analysis). It is shown that our method had better performance than other methods in identifying DMRs when there are high heterogeneity and correlation characteristics of methylation data.

### 3.2. Real Data Application

The real data we used is breast cancer (BRCA) which is available at TCGA. Preprocessing of DNA methylation data is implemented with reference to [35]. We permute sample labels 500 times and calculate the permuted energy for each site for each time. The value in descending order of 5% is obtained for each time and *τ* is obtained averaging these 500 values.

To compare the performance of our method, we apply the method and Wang's method to DNA methylation based on *β*-value and* M*-value, respectively, in our experiment. The thresholds *τ* are set as -2 and -5 for *β*-value and* M*-value data, respectively. The results show that our method can identify more DMRs based on either *β*-value or* M*-value data with a high overlapping ratio (see Table 4), especially that based on* M*-values. Therefore, we implement the next analysis based on the results of* M*-values data.

Based on* M*-value, we also compare our method with DMRcate [28] and ProbeLasso [29] and calculate the overlap of different methods (see Table 5). It is shown that our method has more common DMRs than any of the other methods.

**Figure 2:**
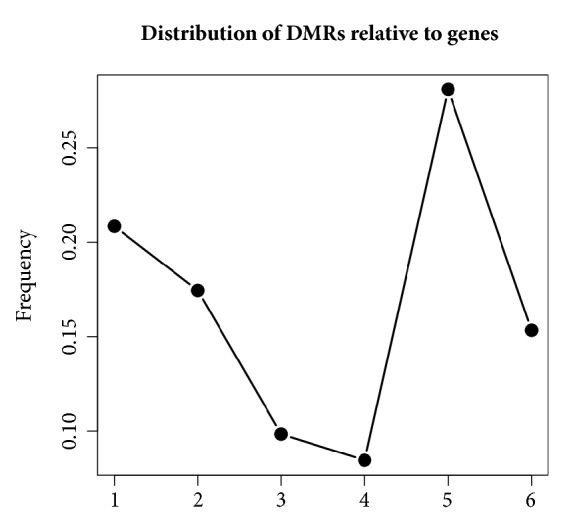
Distribution of DMRs relative to genes.

**Figure 3:**
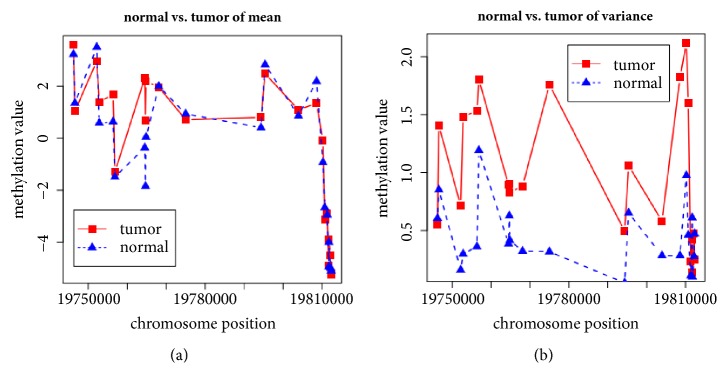
An example of DMRs identified based on* M*-value but not for *β*-value. The difference of these two DMRs in mean signal (a) and variance signal (b) between normal and tumor samples.

Through analyzing the location in genome of these DMRs, we find that about 90% DMRs (7081 in 7871) are located in CGI which are CpG enrichment regions. This is consistent with the fact that aberrant methylation in CGI always influences the expression of genes. To understand the possible functions of DMRs, we evaluate the enrichment of these DMRs according to the location relative to genes (see Figure 2; numbers 1 to 6 represent different regions TSS1500, TSS200, 5'UTR, 1stExon, gene body, and 3'UTR, respectively). It is shown that most DMRs are enriched in gene body which is consistent with the recent studies reporting that aberrant methylation in gene body has essential role in cancer occurrence and development [39]. One example which is identified based on* M*-value but not for *β*-value is shown in Figure 3. It has obvious significant difference in variance signal but not in mean signal.

### 3.3. Validation of Identified DMRs

To illuminate the necessity of integrating the characteristics of DNA methylation data, we compare the results when only considering one signal (mean or variance) and without considering relationship of neighboring sites, respectively. Firstly, we calculate the energy of each site in (3) when *λ* = 1 (only mean signal) and when *λ* = 0 (only variance signal), respectively, and identify DMRs. Secondly, we do not consider the correlation of neighboring sites; the energy of each site is calculated when *e*_*i*_^*mean*^ = *s*_*i*_^*mean*^ and *e*_*i*_^*var*^ = *s*_*i*_^*var*^in (3).

Take chromosome 1 as an example, the identified DMRs are shown in Table 6. It is shown that there is prominence in identifying DMRs when integrating mean, variance, and correlation characteristics more than when only considering variance signal (*λ*=0) and not considering correlation characteristic. Although there are more DMRs when *λ*=1 than those of integrated method, most of these DMRs contain fewer number of sites. Therefore, we think that the mean signal may be more effective in identifying differentially methylated loci than DMRs.

## 4. Conclusions

In this paper, we proposed a data-driven method to identify DMRs through integrating the characteristics of methylation data. Simulation study has shown that our method is more sensitive than the two alternative methods. Through application to real data, we compared the results of DMRs identified based on *β*-value and* M*-value, respectively, and our method showed further better performance than Wang's method. Based on* M*-value data, the necessity of integrating all characteristics of data is shown through comparing the DMRs identified by different measures. It is also shown that the integration of multiple information is effective in identifying DMR.

Currently, the available DMR identification methods are insufficient in integrating data characteristics. Most methods only consider mean signal, and high heterogeneity of methylation data is not considered. The recent work by Wang et al. is developed through accounting for high heterogeneity, and they obtained some meaningful results. Therefore, integrating more information to identify DMR is required.

Although our method integrates the characteristics of high heterogeneity and correlation of neighbor sites of methylation data, we only consider the correlation of the two neighbors of the site limited by the Ising model. Therefore, first, we hope to integrate more comprehensive information based on biological a priori knowledge to build an appropriate model in the further work; second, in view of the strong relationships of CpG sites, we hope to identify DNA methylation patterns based on building methylation network which has been widely used in identification of disease-related genes [40–43].

## Figures and Tables

**Table 1:** Confusion matrix.

		Identified results of a method	
		Number of differential methylated sites within DMRs	Number of non-differential methylated sites without in DMRs
Real DMRs	Number of differential methylated sites within DMRs	*d*	*c*
		True positive	False negative
	Number of non-differential methylated sites without in DMRs	*b*	*a*
		False positive	True negative

**Table 2:** Different parameter settings.

Parameters	*μ*_1_	*μ*_2_	*v*	*σ*	*ρ*
1	-2	2	1.5	0.3	0.7

2	-2	2	2.5	0.3	0.7

3	-3	3	1.5	0.3	0.7

4	-3	3	2.5	0.3	0.7

5	-2	2	1.5	0.3	0.4

**Table 3:** Comparison of different methods in different parameters.

Parameters	Bump hunting				DMRcate				ProbeLasso				Wang's method				**Our method**			
	SP	SE	No. TP (FP)^a^	No. TP (FP)^b^	SP	SE	No. TP (FP)^a^	No. TP (FP)^b^	SP	SE	No. TP (FP)^a^	No. TP (FP)^b^	SP	SE	No. TP (FP)^a^	No. TP (FP)^b^	SP	SE	No. TP (FP)^a^	No. TP (FP)^b^
1	0.99	0.12	0(1)	0(1)	1.00	0.25	2(0)	2(0)	1.00	0.16	3(1)	1(3)	0.99	0.53	6(1)	3(4)	0.99	0.81	9(0)	7(2)
2	1.00	0.23	2(0)	0(2)	1.00	0.25	2(0)	2(0)	1.00	0.12	2(1)	1(2)	0.99	0.38	5(0)	3(2)	0.99	0.90	10(0)	10(0)
3	1.00	0.15	0(2)	0(2)	1.00	0.25	2(0)	2(0)	1.00	0.16	3(1)	1(3)	1.00	0.59	9(0)	4(5)	0.99	0.82	8(0)	6(2)
4	1.00	0.26	1(1)	2(0)	1.00	0.25	2(0)	2(0)	1.00	0.16	3(1)	1(3)	1.00	0.52	5(0)	3(2)	0.99	0.93	10(0)	10(0)
5	1.00	0.18	3(0)	1(2)	1.00	0.25	2(0)	2(0)	1.00	0.12	2(1)	1(2)	1.00	0.45	8(1)	3(6)	0.99	0.79	9(1)	7(3)

^a^*θ* = 0.2;  ^b^*θ* = 0.5

**Table 4:** Comparison results of our method with Wang's method based on *β*-value and M-value.

	Our method	Wang's method	Overlapping ratio
*β*-value	2127	1618	78.9% (1276/1618)

M-value	7871	3070	93.0% (2856/3070)

Overlapping ratio	88.3% (1879/2127)	98.6% (1595/1618)	- - -

**Table 5:** Overlap results of different methods in identifying DMRs.

ProbeLasso	**6720**	1566	2932	*9077*
DMRcate	**6097**	2579	*7577*	2932
Wang's	**2856**	*3070*	2579	1566
**Our**	***7871***	**2856**	**6097**	**6720**

	**Our**	Wang's	DMRcate	ProbeLasso

*∗* Italic numbers indicate the numbers of DMRs identified by different methods. Black ones are overlap numbers of two methods.

**Table 6:** A Comparison of the results to show the necessity of integrating data characteristics.

	Integrated method	*λ* = 1	*λ* = 0	Without considering correlation
Numbers of DMRs	883	2374	370	34

Overlapping (a/b)^*∗*^	100% (883/883)	64.5% (1532/2374)	100% (370/370)	94.1% (32/34)

*∗*a is the numbers of overlapping DMRs compared with integrated method (1532>883 means that more DMRs coincide with one DMR identified by integrated method.); b is the numbers of DMRs identified by the corresponding method.

## Data Availability

The real data we used is breast cancer (BRCA) which is available at TCGA (https://cancergenome.nih.gov/).

## Supplementary Materials

Supplementary Materials More simulated studies through changing the values of parameters are described in Tables S1-S3.
