# Supplementary material for: Data-Driven-Based Approach to Identifying Differentially Methylated Regions Using Modified 1D Ising Model
Source: Biomed Res Int. 2018 Nov 18;2018:1070645. doi: 10.1155/2018/1070645 (PMC6276520; doi:10.1155/2018/1070645)
Supplement: Supplementary Materials — More simulated studies through changing the values of parameters are described in Tables S1-S3. [file 1070645.f1.docx]

**Simulated studies**

To show the performance of different methods in different parameter settings, we calculate the sensitivity, specificity and numbers of true positive and false positive identified by different methods based on the simulated data. For each parameter, the results at different values of this parameter are calculated when we fix other parameters.

1. The parameter $\alpha$ which describes variance signal is changing from 1.5 to 4.5 by 0.3 and other parameters is fixed as $\sigma\text{=}0.3$, $\rho=0.7$, $\mu_{a}=-3$ and $\mu_{b}=3$. Also, we set $\beta$ as 0.2 loosely. The results are shown as Table S1. It is shown that our method is higher sensitivity than other two methods, especially there are strong variance signal in simulated data.

Table S1 Comparison of different methods in different values of $v$

| $v$ | Bumphunting | | | | Wang’s method | | | | | Our method | | | |
| --- | --- | --- | --- | --- | --- | --- | --- | --- | --- | --- | --- | --- | --- |
|  | SP | SE | No. TP (FP)^a^ |  | | SP | SE | No. TP (FP)^a^ |  | SP | SE | No. TP (FP)^a^ |  |
| 1.5 | 1.00 | 0.35 | 6(1) |  | | 1.00 | 0.42 | 3(5) |  | **0.99** | **0.67** | **7(0)** |  |
| | 1.8 | | --- | | 1.00 | 0.12 | 2(0) |  | | 1.00 | 0.41 | 3(3) |  | **0.99** | **0.89** | **10(1)** |  |
| | 2.1 | | --- | | 1.00 | 0.12 | 2(0) |  | | 1.00 | 0.35 | 3(2) |  | **0.99** | **0.96** | **10(1)** |  |
| | 2.4 | | --- | | 1.00 | 0.12 | 2(0) |  | | 1.00 | 0.35 | 3(2) |  | **0.99** | **0.98** | **10(0)** |  |
| | 2.7 | | --- | | 1.00 | 0.12 | 2(0) |  | | 1.00 | 0.35 | 3(2) |  | **0.99** | **0.98** | **10(0)** |  |
| 3.0 | 1.00 | 0.12 | 2(0) |  | | 1.00 | 0.35 | 3(2) |  | **0.99** | **0.98** | **10(0)** |  |
| 3.3 | 1.00 | 0.12 | 2(0) |  | | 1.00 | 0.35 | 3(2) |  | **0.99** | **1.00** | **10(0)** |  |
| 3.6 | 1.00 | 0.12 | 2(0) |  | | 1.00 | 0.35 | 3(2) |  | **0.99** | **1.00** | **10(0)** |  |
| 4.0 | 1.00 | 0.12 | 2(0) |  | | 1.00 | 0.35 | 3(2) |  | **0.99** | **1.00** | **10(0)** |  |
| 4.3 | 1.00 | 0.12 | 2(0) |  | | 1.00 | 0.35 | 3(2) |  | **0.99** | **1.00** | **10(0)** |  |

1. The parameter $\mu$ which describes mean signal is changing from 1.5 to 4 by 0.4 ($\mu_{a}=-\mu$, $\mu_{b}=\mu$) and other parameters is fixed as $\sigma\text{=}0.3$, $\rho=0.7$ and $\alpha=1.5$. Also, we set $\beta$ as 0.2 loosely. The results are shown as Table S2. It is shown that all of methods have high false positive rate when $\mu=1.5$. The reason may be that the mean signal of embed DMRs is small and it is not significantly different from the random ones. Our method has a better performance than other tow method as the mean signal intensifying.

Table S2 Comparison of different methods in different values of $\mu$

| $\mu$ | Bumphunting | | | | Wang’s method | | | | | Our method | | | |
| --- | --- | --- | --- | --- | --- | --- | --- | --- | --- | --- | --- | --- | --- |
|  | SP | SE | No. TP (FP)^a^ |  | | SP | SE | No. TP (FP)^a^ |  | SP | SE | No. TP (FP)^a^ |  |
| 1.5 | 1.00 | 0.04 | 0(1) |  | | 1.00 | 0.39 | 2(5) |  | **1.00** | **0.55** | **2(7)** |  |
| | 1.9 | | --- | | 1.00 | 0.08 | 2(0) |  | | 1.00 | 0.35 | 3(2) |  | **1.00** | **0.70** | **8(2)** |  |
| | 2.3 | | --- | | 1.00 | 0.11 | 2(0) |  | | 1.00 | 0.35 | 3(2) |  | **1.00** | **0.70** | **8(2)** |  |
| | 2.7 | | --- | | 1.00 | 0.11 | 2(0) |  | | 1.00 | 0.41 | 3(3) |  | **1.00** | **0.73** | **8(2)** |  |
| | 3.1 | | --- | | 1.00 | 0.11 | 2(0) |  | | 1.00 | 0.41 | 3(3) |  | **1.00** | **0.73** | **8(2)** |  |
| 3.5 | 1.00 | 0.11 | 2(0) |  | | 1.00 | 0.41 | 3(3) |  | **1.00** | **0.73** | **8(2)** |  |
| 3.9 | 1.00 | 0.11 | 2(0) |  | | 1.00 | 0.41 | 3(3) |  | **1.00** | **0.73** | **8(2)** |  |

1. The parameter $\rho$ which describes correlation of neighbor sites is changing from 0.2 to 0.9 by 0.1 and other parameters is fixed as $\sigma\text{=}0.3$, $\alpha=1.5$, $\mu_{a}=-2$ and $\mu_{b}=2$. Also, we set $\beta$ as 0.2 loosely. The results are shown as Table S3.

Table S3 Comparison of different methods in different values of $\rho$

| $\rho$ | Bumphunting | | | | Wang’s method | | | | | Our method | | | |
| --- | --- | --- | --- | --- | --- | --- | --- | --- | --- | --- | --- | --- | --- |
|  | SP | SE | No. TP (FP)^a^ |  | | SP | SE | No. TP (FP)^a^ |  | SP | SE | No. TP (FP)^a^ |  |
| 0.2 | 1.00 | 0.15 | 4(2) |  | | 1.00 | 0.54 | 5(7) |  | **0.999** | **0.76** | **7(2)** |  |
| | 0.3 | | --- | | 1.00 | 0.08 | 2(0) |  | | 1.00 | 0.35 | 3(2) |  | **0.999** | **0.70** | **8(2)** |  |
| | 0.4 | | --- | | 1.00 | 0.08 | 2(0) |  | | 1.00 | 0.35 | 3(2) |  | **0.999** | **0.70** | **8(2)** |  |
| | 0.5 | | --- | | 1.00 | 0.08 | 2(0) |  | | 1.00 | 0.35 | 3(2) |  | **0.999** | **0.70** | **8(2)** |  |
| | 0.6 | | --- | | 1.00 | 0.08 | 2(0) |  | | 1.00 | 0.35 | 3(2) |  | **0.999** | **0.70** | **8(2)** |  |
| 0.7 | 1.00 | 0.08 | 2(0) |  | | 1.00 | 0.35 | 3(2) |  | **0.999** | **0.70** | **8(2)** |  |
| 0.8 | 1.00 | 0.08 | 2(0) |  | | 1.00 | 0.35 | 3(2) |  | **0.999** | **0.70** | **8(2)** |  |
| 0.9 | 1.00 | 0.08 | 2(0) |  | | 1.00 | 0.35 | 3(2) |  | **0.999** | **0.70** | **8(2)** |  |

It is shown that although our method has higher true positive rate than other two method, all of methods have no change when changing $\rho$ from 0.3 to 0.9. To explain the phenomenon, we test the results at the parameters with high variance signal and strong correlation characteristic ($\alpha=3.5$, $\rho=0.9$). The sensitivities of Bumphunting, Wang’s method and our method are 0, 0.55 and 0.97 respectively. The numbers of true positive (false positive) are 0(0), 4(8), 10(1) respectively. Therefore, our method is considered to have better performance in identify DMRs.
